# Prompt Engineering in Clinical Practice: Tutorial for Clinicians

**DOI:** 10.2196/72644

**Published:** 2025-09-15

**Authors:** Jialin Liu, Fang Liu, Changyu Wang, Siru Liu

**Affiliations:** 1 Department of Medical Informatics, West China Hospital, Sichuan University, Chengdu, China; 2 Department of Otolaryngology - Head and Neck Surgery, West China Hospital, Sichuan University, Chengdu, China; 3 Department of Nephrology, West China Hospital, Sichuan University, Chengdu, China; 4 Laboratory of Diabetic Kidney Disease, Kidney Research Institute, Department of Nephrology, West China Hospital, Sichuan University, Chengdu, China; 5 School of Physical & Mathematical Sciences, Nanyang Technological University, Singapore, Singapore; 6 Department of Biomedical Informatics, Vanderbilt University Medical Center, 2525 West End Ave, Nashville, TN, 37215, United States, 1 615-936-6867

**Keywords:** prompt engineering, large language model, GPT, human-AI collaboration, clinical practice

## Abstract

Large language models (LLMs), such as OpenAI’s GPT series and Google’s PaLM, are transforming health care by improving clinical decision-making, enhancing patient communication, and simplifying administrative tasks. However, their performance relies heavily on prompt design, as small changes in wording or structure can greatly impact output quality. This presents challenges for clinicians who are not experts in natural language processing (NLP). This tutorial combines prompt engineering techniques tailored for clinical use, covering methods like zero-shot prompting, one-shot prompting, few-shot prompting, chain-of-thought prompting, self-consistency prompting, generated knowledge prompting, and meta-prompting. We provide actionable guidance on defining objectives, applying core principles, iterative prompt refinement, and integration into interoperable electronic health record (EHR) systems. This framework helps clinicians leverage LLMs to improve decision-making, streamline documentation, and enhance patient communication while maintaining ethical standards and ensuring patient safety.

## Introduction

Large language models (LLMs), including OpenAI’s GPT series and Google’s PaLM, have catalyzed transformative changes in health care by enabling applications such as clinical decision support (CDS), patient education, and administrative automation [1-3]. Trained on vast datasets, these models generate human-like text, translate languages, and address complex queries. LLMs show significant potential in health care [4], including generating discharge summaries, supporting differential diagnoses, and enhancing patient communication [2,5-10], even though they were not originally designed for medical tasks.

The effectiveness of LLMs hinges on the quality of prompts, that is, crafted instructions that guide model outputs [11]. Minor changes in prompt wording, structure, or tone can lead to marked variability in output relevance and accuracy, underscoring the importance of prompt engineering [12,13]. In clinical settings, where precision is critical, poorly designed prompts can result in generic, inaccurate, or biased outputs, hindering adoption by clinicians unfamiliar with artificial intelligence (AI) or natural language processing (NLP). To address this, researchers have developed techniques like zero-shot, few-shot, and chain-of-thought prompting to optimize LLM performance [12-15]. However, these methods often lack consistency across clinical applications and may not fully address LLM limitations, such as handling rare diseases or integrating multimodal data (eg, text and imaging) [16].

To address these challenges, this study provides a comprehensive, clinician-focused guide to prompt engineering for LLMs in clinical practice. Our objectives are to: (1) catalog clinically relevant prompting approaches and clarify their performance determinants; (2) analyze challenges (accuracy, bias, privacy, and workflow integration) through practical scenarios; and (3) propose actionable recommendations for designing, validating, and integrating prompts into clinical workflows, ensuring safety, equity, and compliance with regulations like the Health Insurance Portability and Accountability Act (HIPAA) and the General Data Protection Regulation (GDPR).

## Define the Clinical Objective

Effective prompt engineering requires a clear clinical objective. Common goals include generating discharge summaries, providing differential diagnoses, creating patient education materials, or supporting treatment planning. Clinicians should consider:

1. Task: what is the specific clinical task (eg, summarizing a patient’s medical history, recommending diagnostic tests)?
2. Audience: who is the intended recipient (eg, clinician, patient, and administrative staff)?
3. Output: what format is required (eg, structured summary, narrative explanation, and list of recommendations)?
4. Example: a clinician aims to generate a discharge summary for a 60-year-old male hospitalized with community-acquired pneumonia, including hospital course, discharge medications, and follow-up instructions, according to 2019 American Thoracic Society and Infectious Diseases Society of America (2019 ATS and IDSA) guidelines.

## Key Principles for Clinical Prompt Engineering

**Figure 1.**
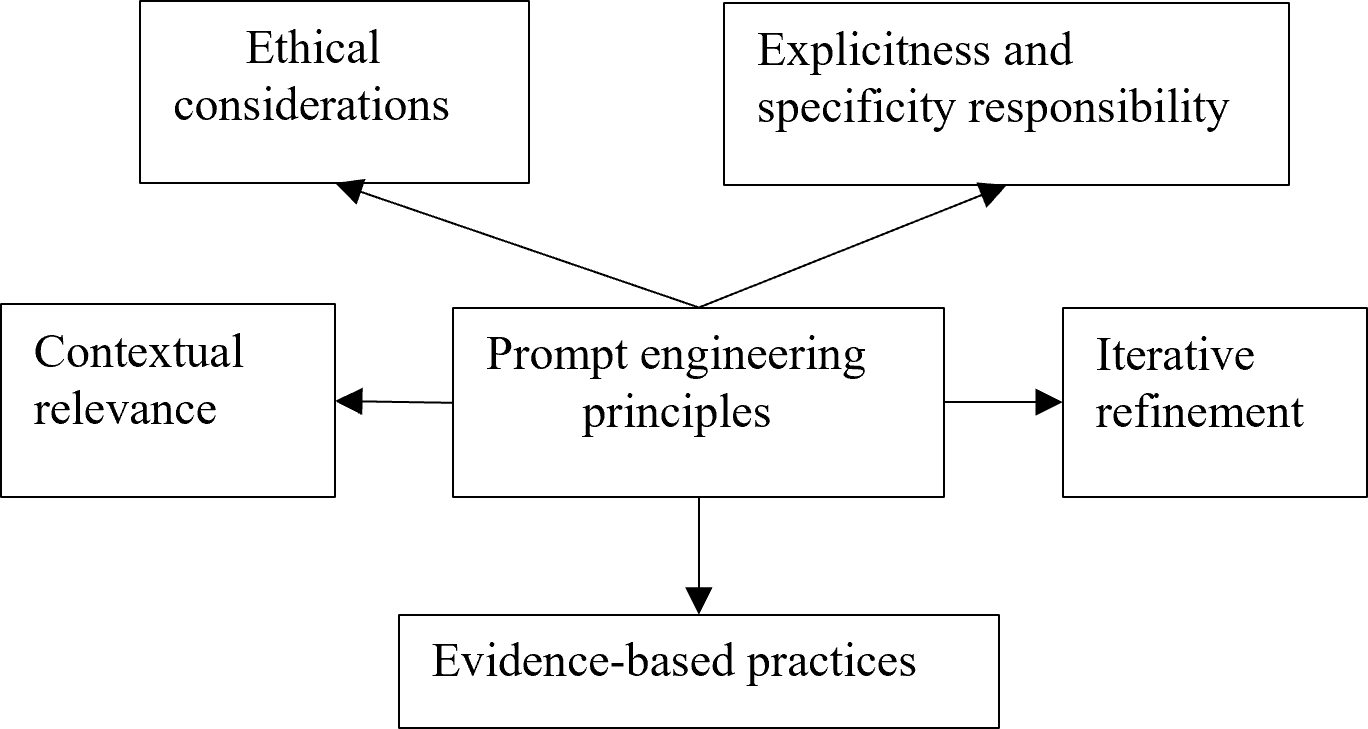
Key principles for clinical prompt engineering.

Based on a comprehensive literature review [11,14,17-24] and our clinical practice, we have identified 5 key principles for effective prompt engineering in clinical settings (Figure 1): explicitness and specificity, contextual relevance, iterative refinement, ethical considerations, and evidence-based practices. These principles enable clinicians to design prompts that optimize LLMs for accurate, relevant, and safe outputs.

### Explicitness and Specificity

Prompts must be clear, precise, and concise to elicit high-quality responses [20]. This is because vague or overly general prompts often result in nonspecific or clinically irrelevant outputs. Clinicians should therefore leverage medical expertise to embed clinically relevant variables, such as patient demographics, comorbidities, and applicable guidelines.

For example, instead of asking, “What are the treatment options for diabetes?,” consider: “For a 50-year-old male with type 2 diabetes, ischemic heart disease, and stage 3 chronic kidney disease (CKD) (estimated glomerular filtration rate: eGFR 50 ml/min/1.73m²), who cannot tolerate metformin, what are the recommended pharmacological alternatives per 2023 ADA and KDIGO guidelines? Compare the renal and cardiovascular benefits of sodium-glucose cotransporter 2 (SGLT2) inhibitors and glucagon-like peptide-1 (GLP-1) receptor agonists for this profile.”

A strategy would be to incorporate patient-specific clinical attributes (eg, age, disease stage, and comorbid conditions) and reference updated clinical guidelines to reduce ambiguity and improve the clinical validity of generated outputs.

### Contextual Relevance

Incorporating relevant clinical details into prompts directly impacts the quality of LLM responses. The more contextually relevant the information provided, the more specific and accurate the generated outputs will be [21]. Clinicians must evaluate which clinical details are most pertinent by analyzing the situation and emphasizing key facts.

For example, “For a 70-year-old male with hypertension and diabetes, considered for laryngeal cancer surgery, outline risks, benefits, and evidence-based alternatives per National Comprehensive Cancer Network (NCCN) guidelines.”

A strategy would be to incorporate patient history and clinical context by embedding prompts within electronic health records (EHRs) using Substitutable Medical Applications and Reusable Technologies (SMART) on Fast Healthcare Interoperability Resources (FHIR) to generate tailored responses.

### Iterative Refinement

Iterative refinement is a critical strategy when using LLMs in clinical practice, as initial outputs may be incomplete, overly general, or misaligned with the clinical context [21,22]. Clinicians should revise the prompt by adjusting its phrasing, increasing its clinical specificity, or adding contextual details to enhance the relevance and accuracy of subsequent responses.

For example, if the initial prompt “What are the treatment options for diabetes?” yields overly generic recommendations. It should be refined to: “According to the 2023 ADA guidelines, what are the recommended pharmacologic treatments for a 65-year-old patient with type 2 diabetes and stage 3 CKD (eGFR 45 mL/min/1.73 m²) who is intolerant to metformin?”

The core strategy is to implement a structured feedback loop driven by clinical judgment. This involves assessing each AI response for its completeness, alignment with guidelines, and clinical plausibility for the specific patient. To ensure rigor and traceability, clinicians can document key prompt iterations and their corresponding outputs, tracking how refinements improve alignment with therapeutic goals.

### Ethical Considerations

When crafting prompts, clinicians must prioritize patient privacy and adhere to ethical standards, particularly when using consumer-grade LLMs (eg, ChatGPT) that are not designed for secure health care environments. A deep understanding of clinical ethics is essential to ensure responsible and equitable AI integration in practice [23].

An example prompt would be, “A 65-year-old male with heart failure with reduced ejection fraction (EF 35%) and a history of hypertension is eligible for a new heart failure medication, but the drug is not covered by his insurance. What are guideline-recommended, cost-effective alternatives? Consider barriers to medication access and patient adherence.”

Strategies for this purpose include deidentification, which involves using techniques such as k-anonymity to anonymize patient data and comply with the HIPAA and GDPR regulations; bias mitigation, which requires monitoring outputs for demographic biases using fairness metrics, such as disparate impact ratios, and validating against diverse patient cohorts; and bioethical alignment, which incorporates the 4 principles of medical ethics (autonomy, beneficence, nonmaleficence, and justice) into prompt design to ensure fairness and safety in AI-assisted care [23] .

### Evidence-Based Practices

Prompts should be designed to align LLM-generated outputs with the latest clinical guidelines and evidence-based practices to ensure scientific validity [11]. Clinicians can instruct LLMs to summarize recent studies or extract key recommendations from updated guidelines (eg, ACC, AHA, and NICE) for diagnostic or therapeutic decisions. Given the potential for hallucinations or outdated content, all LLM outputs should then be verified by cross-checking with primary literature and authoritative databases such as UpToDate or PubMed.

For example, “A 65-year-old patient with stable coronary artery disease is currently on aspirin. Summarize the latest evidence from post-2021 trials (eg, The Lancet) on continuing aspirin for primary versus secondary prevention, and cite any updates from the 2023 ACC and AHA guidelines.”

As a strategy, it is recommended to request guideline-linked responses and verify outputs through trusted, regularly updated medical databases to ensure both clinical relevance and currency. By mastering these principles, clinicians can shift from passive interaction with AI to actively designing precise, evidence-aligned queries enhancing the reliability, safety, and fairness of LLMs in practice.

## Choose a Prompting Technique

Prompting techniques are structured methods for crafting single or sequenced instructions to LLMs [25,26]. These methods range from incorporating dynamic features, such as conditional or branching logic that adapts to user input, to using advanced architectural strategies [14,24,26]. Such strategies, including parallel prompting and multiprompt integration, enable simulation of clinical workflows [27]. The most common techniques and their clinical applications, strengths, and limitations are summarized in Table 1 [25, 28-60].

**Table 1.** Prompting techniques for clinical applications.

Technique	Clinical application	Strengths	Limitations
Zero-shot	General queries, discharge summaries	Flexible, requires no examples, ideal for quick queries.	May produce generic outputs
One-shot	Patient education, standardized notes	Minimal effort for structured outputs, reduces overfitting risk	Limited guidance for complex tasks; output quality depends on the example’s relevance
Few-shot	Diagnostic support, documentation	Enhances output consistency and relevance for complex tasks	Requires curated examples, increasing setup time; risk of overfitting to provided examples
Chain-of-thought	Differential diagnosis, complex cases	Improves reasoning for complex cases,	May generate verbose outputs
Self-consistency	Diagnostic support, risk stratification	Reduces output variability, increases confidence in recommendations	Computationally intensive, requires validation to avoid consistent but incorrect outputs
Generated knowledge	Guideline-based recommendations	Enhances context for complex queries, supports tailored responses	Risk of hallucination; complexity hinders routine use
Meta-prompting	Prompt optimization	Systematically improves prompt quality	Requires expertise to design meta-prompts, time-intensive for routine use.

### Zero-Shot Prompting

Zero-shot prompting leverages an LLM’s pretrained knowledge without examples, offering flexibility for general queries [28].

Steps: (1) draft a concise, specific instruction defining the clinical task, (2) embed patient details (eg, age, symptoms, and comorbidities), and (3) review output for accuracy and completeness.

Example:

1. Task: draft a discharge summary.
2. Prompt: *“S*ummarize the medical history and treatment plan for a 60-year-old male with chronic obstructive pulmonary disease (COPD) and hypertension, admitted for 5 days with community-acquired pneumonia, treated with ceftriaxone and azithromycin, now stable with SpO₂ 95%.”
3. Output: a succinct hospital course summary with tailored discharge instructions.
4. Strengths: requires no examples, ideal for quick queries [28,31].
5. Limitations: may produce generic outputs for complex tasks; reliant on pretrained knowledge [28,31].

### One-Shot Prompting

One-shot prompting provides a single exemplar to guide output format or reasoning [32]. The exemplar serves as a template, guiding the model in understanding the desired output format or task requirements.

Steps: (1) define the desired output format (eg, patient education leaflet). (2) Include one example in the prompt. (3) Add the specific clinical query. (4) Refine output if needed.

Example:

1. Task: create a patient education leaflet for hypertension.
2. Prompt: “Example: ‘Diabetes is a condition where your body struggles to control blood sugar due to insufficient insulin or poor insulin use, leading to health risks if unmanaged.’ Now, explain hypertension in a similar patient-friendly style.”
3. Output: a clear, concise explanation of hypertension.
4. Strengths: minimal effort for structured outputs [33,34].
5. Limitations: limited guidance for highly variable tasks [33,34].

### Few-Shot Prompting

Few-shot prompting uses 2‐5 structured examples to align an LLM’s output with specific clinical tasks [34-37]. This approach improves both consistency and contextual accuracy.

Steps: (1) curate a diverse set of task-relevant examples. (2) Embed the examples sequentially in the prompt, followed by the main query. (3) Assess the final output for accuracy, coherence, and clinical relevance.

Example:

1. Task: predict likely diagnoses.
2. Prompt: “Example 1: A 45-year-old male with fever, productive cough, and shortness of breath (Saturation of peripheral oxygen: SpO_2_ 92%)→Community-acquired pneumonia. Example 2: A 30-year-old female with headache, neck stiffness, and fever (38.5 °C)→Bacterial meningitis. Example 3: A 60-year-old male with fatigue, unintentional 10% weight loss over 6 months, and persistent cough→Pulmonary tuberculosis. For a 32-year-old pregnant female (28 weeks of gestation) with high blood pressure (150/95 mm Hg), proteinuria (2+ on dipstick), and bilateral lower extremity edema→?”
3. Output: a ranked list of diagnoses (eg, preeclampsia, CKD, and gestational hypertension) with rationale according to the 2023 ACOG (American College of Obstetricians and Gynecologists) guidelines.
4. Strengths: Improves consistency in complex tasks through clear example prompts [34-37].
5. Limitations: Time-intensive curation; effectiveness depends on example quality and relevance [34,38].

### Chain-of-Thought (COT) Prompting

COT prompting is a technique that elicits explicit reasoning from LLMs by incorporating step-by-step reasoning demonstrations or instructions within the prompt [39]. This approach mimics the human method of problem solving, where individuals solve complex problems through step-by-step reasoning [39,40]. COT prompting increases transparency, helps avoid errors, and leads to more informed decisions [39-41].

Steps: (1) request explicit reasoning steps in the prompt. (2) Include clinical details and specify the reasoning process. (3) Validate output for logical coherence and clinical accuracy.

Example:

1. Task: identify diagnoses for chest pain.
2. Prompt: “For a 50-year-old male with 2-day chest pain and shortness of breath, list possible diagnoses. First, rule out life-threatening conditions (eg, acute coronary syndrome, pulmonary embolism), then consider secondary causes (eg, pneumonia, gastroesophageal reflux disease). Specify key features and tests for each.”
3. Output: a structured list of diagnoses with reasoning and tests.
4. Strengths: improves reasoning for complex cases [40,41].
5. Limitations: may generate verbose outputs, requiring validation [42-44].

### Self-Consistency Prompting

Self-consistency prompting is a technique that improves response reliability by generating multiple iterations and selecting the most consistent output [41]. In this technique, an LLM generates multiple responses to the same prompt and then evaluates or compares these responses to select the most consistent, reasonable, or accurate answer [30,41].

Steps: (1) craft one detailed prompt with all relevant patient data. (2) Submit it 3‐5 times to generate independent outputs. (3) Identify the majority consensus or stable pattern and validate it against clinical guidelines and expert judgment.

Example:

1. Task: assess the 30-day readmission risk for a high-risk patient with heart failure.
2. Prompt: a 75-year-old female with a history of ischemic cardiomyopathy (EF 30%), stage 3 CKD, diabetes, and a prior admission for decompensated heart failure 2 months ago, assess her 30-day readmission risk as low, moderate, or high. Provide step-by-step reasoning for your assessment.
3. Output: 4 of 5 runs indicate high risk, increasing confidence in this assessment.
4. Strengths: improves robustness in probabilistic tasks and reduces random output variability [45,46].
5. Limitations: more computationally intensive; consensus may still reflect model bias and requires clinician verification [45-47].

### Generated Knowledge Prompting (GKP)

GKP is a 2-stage technique where the LLM first generates relevant factual knowledge about a topic and then applies this knowledge to address a specific clinical question [48]. By explicitly eliciting the model’s parametric knowledge, GKP enhances performance on knowledge-intensive tasks, such as diagnostic reasoning or treatment planning [49,50]. In clinical practice, GKP can produce comprehensive background information (eg, disease pathophysiology, current guidelines) before answering targeted diagnostic or therapeutic queries, thereby improving reasoning accuracy and depth [51-53].

Steps: (1) Elicit knowledge: prompt the LLM to summarize relevant medical facts. (2) Apply knowledge: use the generated summary to frame a specific clinical question. (3) Validate: cross-check outputs against evidence-based guidelines or primary literature (eg, UpToDate and PubMed).

Example:

1. Task: identify causes of acute chest pain.
2. Prompts: “Summarize the major causes of acute chest pain in adults, including cardiac, pulmonary, gastrointestinal, and musculoskeletal etiologies, according to the 2023 ACC and AHA guidelines.” And “Using that summary, list and prioritize causes of acute chest pain for a 60-year-old male with a history of hypertension, smoking (20 pack-years), atypical chest discomfort (3/10 severity, 2-hour duration), normal electrocardiogram (ECG), and negative troponin, according to the 2023 ACC and AHA guidelines.”
3. Output: major etiologies of acute chest pain include acute coronary syndrome, gastroesophageal reflux disease, musculoskeletal chest pain, aortic dissection, and pulmonary embolism [53,54].
4. Strengths: provides richer context for complex clinical queries, reducing omission errors
5. Limitations: added complexity; risk of hallucination requires rigorous validation [52-54].

### Meta-Prompting

Meta-prompting is a prompt engineering technique in which one prompt (“meta-prompt”) is used to evaluate and refine another prompt (“base prompt”), enhancing clarity, scope, and relevance [55-60]. Instead of directly seeking an answer, meta-prompts focus on improving the question’s structure, context, and objectives before final execution [5,6,55]. This higher level of abstraction helps optimize interactions with LLMs by making prompt creation more precise and effective, particularly for clinical tasks [59].

Steps: (1) write an initial base prompt for a clinical task. (2) Craft a meta-prompt to evaluate and optimize the base prompt’s content, structure, and clinical appropriateness. (3) Use the optimized base prompt to generate the final output.

Example:

1. Task: refine a prompt for pneumonia diagnosis.
2. Base prompt: “What are the symptoms and risk factors of pneumonia in a patient with cough and fever?”
3. Meta-prompt output: “The base prompt lacks patient-specific details (eg, age, comorbidities) and guideline references, limiting diagnostic precision.”
4. Optimized prompt: “For a 65-year-old patient with cough and fever, and a history of COPD, list pneumonia symptoms, risk factors, and differential diagnoses, including evidence-based criteria according to 2023 American Thoracic Society and Infectious Diseases Society of America (ATS and IDSA) guidelines.”
5. Output: a comprehensive list of pneumonia symptoms, risk factors, and differential diagnoses tailored to the patient, with diagnostic criteria referenced to 2023 ATS and IDSA guidelines.
6. Strengths: improves prompt quality and relevance systematically [59,60].
7. Limitations: requires domain expertise to craft effective meta-prompts [59,60].

## A Framework for Clinical Prompt Implementation

We propose a comprehensive framework for the clinical implementation of prompts based on extensive research and clinical practice [61,62]. This framework consists of 3 key components: prompt design and validation, output evaluation and optimization, and prompt integration into clinical workflows [62].

### Design and Validate Prompts

Clinicians must design precise, secure prompts and validate them in controlled environments. This ensures clinical relevance and compliance with regulations, such as HIPAA and GDPR.

### Formulate Prompts

Formulate prompts by constructing clear and concise instructions that include patient-specific details and task requirements. For example, “A 58-year-old male with hypertension and diabetes presents with chest pain for two days. List possible diagnoses, prioritize life-threatening conditions (eg, acute coronary syndrome), and recommend tests according to 2023 ACC and AHA guidelines.” The strategy is to include specific clinical details such as risk factors, clinical measurements, and guideline references to ensure the LLM’s response is focused and clinically relevant.

#### Deidentification Prompts

Deidentification prompts should ensure that all inputs are free from personally identifiable information (PII) or protected health information (PHI). Robust methods such as k-anonymity and pseudonymization should be applied, and compliance with HIPAA and GDPR must be documented [63,64]. Future multimodal prompts that integrate images, genomic data, or other sensitive information will require advanced privacy-by-design approaches. A key strategy is to employ automated PII and PHI detection platforms as secondary safeguards.

#### Test Prompts

Test prompts should be executed on HIPAA-compliant platforms, such as Health Information Trust Alliance Common Security Framework (HITRUST CSF)-certified sandboxes or integrated EHR tools (eg, Epic CDS). The strategy is to leverage platforms with automated PII and PHI detection capabilities to ensure secure and compliant use.

#### Validate Outputs

Validate outputs by assessing them for clinical accuracy, relevance, completeness, and equity in alignment with evidence-based standards such as UpToDate and peer-reviewed literature. The strategy is to implement a multireviewer process involving subject matter experts to detect errors, omissions, or potential biases.

### Evaluate and Refine the Output

The initial LLM output should be viewed as a preliminary draft, requiring structured, iterative evaluation and refinement.

The clinical validity of LLM outputs should be evaluated by comparing them against current evidence-based guidelines (eg, ACC, AHA, and NICE) and established clinical knowledge, engaging a multidisciplinary team to identify discrepancies and document errors. In addition, audit for bias and hallucinations by checking for demographic or socioeconomic biases and factually incorrect statements, using fairness metrics and validating claims against authoritative clinical sources such as PubMed; multimodal systems may require new validation paradigms due to cross-data biases. To improve outputs, revise prompts by targeting one deficiency per revision, adding necessary context, or refining instructions with clearly defined corrective constraints. Continue iterating this process until the outputs meet predefined thresholds for safety, accuracy, and equity, using clinical metrics to evaluate and refine each iteration.

### Integrate Prompts Into Clinical Workflows

Integrating LLM prompts into clinical workflows is a complex socio-technical challenge, requiring a robust framework to ensure effectiveness and minimize risks [65].

Opportunities for embedding prompts include using SMART on FHIR or CDS hooks to integrate real-time EHR data at critical touchpoints (eg, order entry). SMART on FHIR enables integration of LLMs with EHRs through standardized APIs, which IT teams can configure using tools like Epic’s App Orchard [66]. To implement this effectively, EHR event triggers should be defined and FHIR resources mapped to deliver relevant prompts without disrupting workflows. To mitigate alert fatigue, design principles should be applied, such as tiered alerting that classifies prompts by clinical urgency, contextual suppression that hides lower-tier alerts when higher-urgency alerts are active, and actionable recommendations that provide specific next steps linked to guideline references [67]. Clinician override rates and feedback should be monitored to calibrate alert tiers and recommendation formats. Controlled environment testing should also be conducted through usability simulations, measuring performance metrics such as alert acknowledgment rate and time-to-action, with Institutional Review Board (IRB) approval required for human participant studies. In addition, Failure Mode and Effects Analysis (FMEA) should be performed to identify and mitigate safety hazards, systematically examining potential failures in prompt-driven workflows—such as incorrect diagnoses—with guidance from quality improvement teams [68]. IRB-approved simulations and FMEA sessions should be deployed to refine system design and workflows.

## Future Directions

As LLMs advance, several research areas will be critical to maximizing their potential in clinical settings. Key directions include:

### Adaptive and Context Aware Prompting

Future prompts should dynamically adapt to clinician intent, patient status, and care context by leveraging: (1) hierarchical context modeling: Use layered data representations (eg, chief complaint→vitals→lab results) to enhance prompt specificity [69]. (2) Multimodal data fusion: real-time synthesis of EHR entries, imaging metadata, and patient history. (3) Reinforcement learning frameworks: Tune prompts based on clinician feedback and clinical outcomes, with safeguards to prevent bias amplification. (4) Standards-based integration: Embed prompts within SMART on FHIR and CDS Hooks workflows at key decision points for seamless interoperability [66].

### Real-Time CDS

In acute and critical care, prompt engineering must deliver immediate, actionable guidance: (1) interoperable application programming interfaces (APIs): support bidirectional, low-latency data exchange between LLM engines and EHR platforms. (2) Middleware orchestration: unify heterogeneous data streams (eg, vitals, lab results, imaging) into a cohesive model for prompt generation. (3) Tiered alerting strategies: classify recommendations by urgency (eg, emergent vs informational) to minimize alert fatigue while ensuring safety. (4) Track metrics like acknowledgment rates, override frequencies, and clinical outcomes to refine prompt content and timing [67].

### Multimodal Prompting

Effective integration of textual and nontextual inputs will require: (1) modality weighting algorithms: prioritize relevant data types (eg, radiographic findings over narrative notes) based on clinical context. (2) Sequential hierarchical prompting: process each data modality sequentially before integration, mirroring clinical reasoning workflows. (3) Confidence-driven adaptation: use uncertainty estimates (eg, Monte Carlo dropout) to trigger follow-up queries or escalate to human review when confidence is low [70].

### Personalization and Accessibility

Tailoring prompts to individual users and diverse patient populations enhances relevance and equity: (1) user-adaptive learning: adapt to clinician preferences (eg, specialty-specific terminology and detail level) to reduce irrelevant alerts. (2) Multilingual and culturally sensitive content: localize prompts to accommodate varied linguistic and cultural backgrounds. (3) Inclusive design: provide alternative modalities (eg, voice-activated prompts and adjustable text size) to support users with sensory or cognitive impairments [71].

## Limitations

Despite grounding this framework and its illustrative cases in real-world clinical practice, several limitations must be acknowledged. (1) Cross-specialty generalizability: while the clinical reasoning cycle (data collection, differential diagnosis, and decision formulation) is consistent across medical disciplines, variations in domain-specific knowledge, data modalities, and guideline frameworks require tailored prompt adaptations. For example, prompts for cardiology, which emphasize hemodynamic parameters and electrophysiological metrics, may need modification to address oncology’s tumor staging criteria or pediatrics’ age-specific developmental norms. (2) Model-dependent performance: the effectiveness of prompt engineering techniques depends on the underlying LLM architecture. Variations in model size, training data, and inference behavior can significantly impact output quality and reliability, necessitating model-specific testing and optimization. (3) Institutional and cultural variability: heterogeneity in clinical workflows, regulatory requirements (eg, HIPAA and GDPR), and cultural norms across health care settings and regions may affect the feasibility and clinician acceptance of prompt-based interventions. For instance, differences in EHR adoption or cultural attitudes toward AI may influence the success of integration.

## Conclusions

This tutorial provides a practical framework for leveraging LLMs through prompt engineering in clinical practice. By defining objectives, applying core principles, selecting techniques, crafting and refining prompts, and embedding them into workflows, clinicians can enhance decision-making, patient communication, and care delivery. With ongoing practice and ethical vigilance, prompt engineering enables personalized, evidence-based care. We encourage clinicians to adopt these techniques, collaborate with informatics teams, and contribute to research on AI integration in health care.

## Abbreviations

ACOG: American College of Obstetricians and Gynecologists
ADA: American Diabetes Association
AI: artificial intelligence
ATS: American Thoracic Society
CDS: clinical decision support
CKD: chronic kidney disease
COT: chain of thought
CSF: Health Information Trust Alliance Common Security Framework
EHR: electronic health record
FHIR: Fast Healthcare Interoperability Resources
FMEA: Failure Mode and Effects Analysis
GDPR: General Data Protection Regulation
GKP: generated knowledge prompting
HIPAA: Health Insurance Portability and Accountability Act
HITRUST CSF: Health Information Trust Alliance Common Security Framework
IDSA: Infectious Diseases Society of America
IRB: Institutional Review Board
LLM: large language model
NCCN: National Comprehensive Cancer Network
NLP: natural language processing
PHI: protected health information
PII: personally identifiable information
SMART: Substitutable Medical Applications and Reusable Technologies
